# Plant Growth Promoting Rhizobacteria in Plant Health: A Perspective Study of the Underground Interaction

**DOI:** 10.3390/plants12030629

**Published:** 2023-01-31

**Authors:** Mudasir Ahmad Bhat, Awdhesh Kumar Mishra, Saima Jan, Mujtaba Aamir Bhat, Mohammad Azhar Kamal, Safikur Rahman, Ali Asghar Shah, Arif Tasleem Jan

**Affiliations:** 1 Department of Biotechnology, School of Biosciences and Biotechnology, Baba Ghulam Shah Badshah University, Rajouri 185234, India; 2 Department of Biotechnology, Yeungnam University, Gyeongsan 38541, Republic of Korea; 3 Gene Expression Lab., School of Biosciences and Biotechnology, Baba Ghulam Shah Badshah University, Rajouri 185234, India; 4 Department of Pharmaceutics, College of Pharmacy, Prince Sattam Bin Abdulaziz University, Alkharj 11942, Saudi Arabia; 5 Department of Botany, Munshi Singh College, BR Ambedkar Bihar University, Muzaffarpur 845401, India

**Keywords:** abiotic stress, PGPR, plant productivity, rhizosphere, agricultural sustainability

## Abstract

Plants are affected by various environmental stresses such as high or low temperatures, drought, and high salt levels, which can disrupt their normal cellular functioning and impact their growth and productivity. These stressors offer a major constraint to the morphological, physiological, and biochemical parameters; thereby attributing serious complications in the growth of crops such as rice, wheat, and corn. Considering the strategic and intricate association of soil microbiota, known as plant growth-promoting rhizobacteria (PGPR), with the plant roots, PGPR helps plants to adapt and survive under changing environmental conditions and become more resilient to stress. They aid in nutrient acquisition and regulation of water content in the soil and also play a role in regulating osmotic balance and ion homeostasis. Boosting key physiological processes, they contribute significantly to the alleviation of stress and promoting the growth and development of plants. This review examines the use of PGPR in increasing plant tolerance to different stresses, focusing on their impact on water uptake, nutrient acquisition, ion homeostasis, and osmotic balance, as well as their effects on crop yield and food security.

## 1. Introduction

The inception of agricultural practices at the interface of human interaction with the environment has merged crop productivity with consistency in the environment [1]. The increase in the human population (projected to be 30% larger by 2030) and decrease in the cultivable land (due to urbanization, industrialization, and increase in the pollution) offer a big challenge in the current day scenario. To this, it is presumed that either area under cultivation should be increased or the productivity of the already cultivable area be increased in order to achieve sustainability in agriculture that can offer a consistent and adequate food supply for the growing population [2]. Enhancement in agricultural productivity to achieve food security for the growing population is greatly influenced by changes in climatic conditions, farming systems, and management techniques [3]. On one side, where food security for the growing population has put forth the need to have a consistent and adequate food supply to meet the demand (almost double), inclement climatic conditions put humans at stake by exacerbating the severity of different stresses that have pronounced effects on crop productivity. In such a scenario, microbial populations inhabiting the rhizosphere (commonly referred to as plant growth-promoting rhizobacteria, PGPR) may offer a solution by assisting plants through the modulation of the developmental activities that affect overall growth and productivity [4]. The microbial populations contribute to a wide range of biological events at the soil root interface including nutrient acquisition and water uptake, maintenance of osmotic balance, and ion homeostasis; thereby helping in the sustenance of crop health and productivity. Additionally, the root-associated microbial population safeguards plants from soil-borne diseases and reinforces stress tolerance to plants in coping with changing climatic conditions such as drought, salinity, etc [5,6].

Considering the knowledge gaps, this study was performed on the available literature of different databases such as PubMed, Web of Sciences, Google Scholar, etc, in order to provide an understanding of the role of the microbiome in assisting the growth of plants under changing climatic conditions. By elucidating the mechanisms of the microbial interactions with plants such as nutrient acquisition, maintenance of ion homeostasis and osmotic balance, regulation of the phytohormone levels, etc, it offers an advantage in mitigating the impact of different environmental factors such as extreme temperature, drought, etc, on productivity and as such improve the resilience of plants to climatic variability. This study covering original research, reviews, opinions, etc, provides adequate information on PGPR functionality and deliberate new ideas and emerging concepts that, if brought to practice, could enhance plant productivity and also make avenues to attain sustainability in agriculture.

## 2. Soil Fertility and Plant Growth

**Figure 1:**
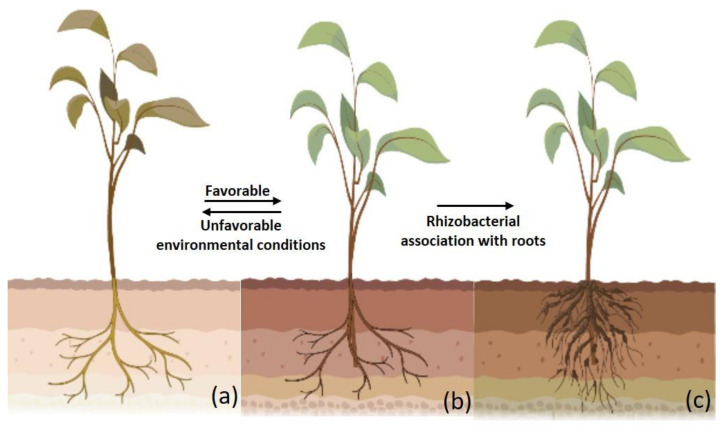
Plant growth under different environmental conditions: (**a**) Progression of climatic conditions from favorable to unfavorable that affects overall performance of the plant, (**b**) Plant growth under normal conditions, and (**c**) Plant-microbiome interactions that affect overall performance of the plant.

Soil is generally considered a non-ideal system owing to its complex chemical and mineral status. An increase in the agricultural productivity for demarked cultivable areas largely depends on soil fertility. Soil fertility refers to the ability of soil to provide essential nutrients in suitable proportions and adequate amounts at the right interval of time to meet the nutritional requirement of the plant in order to sustain its growth [7]. Being a vital and important factor in the determination of productivity (both qualitatively and quantitatively), it is necessary to have regular monitoring of activities such as structural arrangements, holding capacity, transformation ability, and nutrient cycling in a continuous and consistent manner. A decrease in soil fertility offers a major hindrance in achieving sustainability in the production towards fulfilling the need of the growing population (Figure 1). Though the application of inorganic fertilizers complemented the loss in agricultural productivity, its continuous use over the years was found to have adverse effects on soil fertility [8]. In addition to their role in regaining soil fertility, the application of organic fertilizers together with inorganic ones was found effective in increasing agricultural productivity [9].

Despite exerting beneficial effects, enhancement in organic fertilizer usage is directly connected with a change in the biological characteristics of the soil; thereby rating soil fertility and long-term enhancement [10]. The lately emerged eco-friendly biofertilizers, which make use of soil microorganisms to decompose the organic matter to readily available nutrients for plant usage, are considered a better alternative to chemical fertilizers for use in sustainable agricultural practices [11]. Improvising soil biological characteristics causes enhancement in the uptake of mineral nutrients (both micro- and macro) to ameliorate the deficiency in the root region and their uptake within the plant system [12]. The microorganisms exhibit both rhizospheric (*Azospirillum*, *Azotobacter*, *Burkholderia*, *Enterobacter*, *Klebsiella*, *Variovorax*, *Comamonadaceae*, *Pseudomonas*, *Gemmatimonadetes*, *Streptomyces filamentosus*, and *Bacillales*) and endophytic (*Azoarcus* spp., *Herbaspirillum seropedicae*, and *Gluconacetobacter diazotrophicus*) relationship in the intercellular and apoplastic space of the host plants respectively [13,14]. Besides solubilizing mineral nutrients, microorganisms regulate the production of plant growth hormones (such as indoleacetic acid, IAA; cytokinins, CKs; gibberellins, GA) and inhibitors of ethylene [15], support plant growth; thereby resulting in improved overall productivity. 

## 3. Plant Growth Promoting Rhizobacteria

Plants grown in the field represent a complex community with subtle partner association of a well-structured community of microorganisms referred to as phytomicrobiome [16]. The microbiome (predominantly bacteria and fungi) that colonizes the roots (rhizomicrobiome) represents the most popular association at the rhizospheric plane [17]. Of the different microbial populations that inhabit the root surface, a heterogeneous group of bacteria (preferably free living) exerting beneficial effects on plants either directly and/or indirectly are referred to as plant-growth-promoting rhizobacteria (PGPR) [15]. PGPR include both facultative anaerobes that invade intercellular space and thrive as endophytes and those living outside the host plant but are differentially associated with the rhizospheric plane. The PGPR (including rhizospheric and endophytes) induce their beneficial effects by assisting plants in challenging times, either by increasing their access to nutrients or helping them to overcome stressful conditions and pathogens [18].

### 3.1. Root Exudates and PGPR

**Figure 2:**
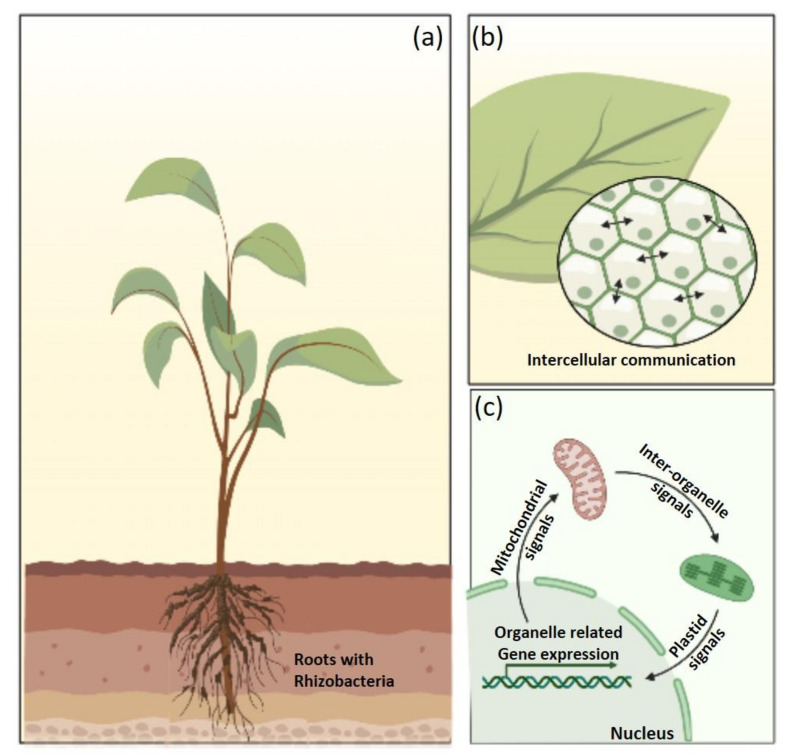
Plant-microbiome interactions: (**a**) PGPR association with roots of the plant, (**b**) Microbial interactions enhancing cellular signaling, and (**c**) Signaling pathway along different modules affecting gene expression and, as such, overall performance of the plant.

Plants provide a suitable niche for the growth and multiplication of a wide variety of microorganisms. Existing in complex communities, the plant-associated microbiota comprising of bacteria, fungi, etc., manifests a series of beneficial traits such as suppression of plant pathogens, transformation and translocation of essential nutrients in the soil for uptake by plants, that promote plant growth and increase productivity [19]. Necessary for establishing a stable plant-microbe interaction, plant root exudates of variable composition regulate the recruitment and composition of the rhizomicrobiome [20]. The root exudates contain a large proportion of amino acids, sugars, and organic acids, and a small number of secondary metabolites such as flavonoids, phenolic compounds, and terpenes [21]. The composition and amount of plant root exudates vary widely depending on plant species and growth stages, which decisively affect the composition and abundance of root-associated microbiota populations [16]. In addition to the secretion of exopolysaccharides and quorum sensing, different substances in the exudate act as signaling molecules that regulate bacterial chemotaxis [22]. Plant root exudation of signaling molecules such as jasmonic acid and salicylic acid regulates the initial events of microbial colonization of plant roots [23]. Acting as an important part of signaling events at the rhizosphere, root exudates under changing conditions and performs inter-organismal communication (Figure 2). Together, molecules secreted from the root support microbial growth and their activity in the rhizosphere, and in return, they help plants in the acquisition of soil resources, impart protection against different pathogens, and promote growth via increased root hair proliferation, and branching, leaf surface area, besides causing an increase in the indigenous hormone levels and enhancement in the accumulation of carbohydrates that improve vigor and biomass right from germination to the full grown and go up to senescence [24].

### 3.2. PGPR in Nutrient Acquisition

Limited nutrient availability and inadequate supply to plants have severe effects on plant growth and productivity. The availability of nutrients in the soil for plants to uptake depends on several parameters such as the composition and moisture content of the soil, its texture, pH, and the existing microflora in the rhizosphere [2]. pH plays an important role in the determination of the sustainability of microbes in the soil, and it is found that the pH ranges from 6.0 to 6.5 favors the beneficiary microbe’s sustainability and ensures healthy plant growth [25]. Though most of the nutrients are available in the pH range of 5–7, their binding to different ions (both cations and anions) in response to changes in the pH makes them less available for uptake by plants. In such a scenario, microbes were found capable of assisting plants in the acquisition of nutrients by employing different mechanisms such as nitrogen fixation, augmentation of surface area accessed by roots, phosphate solubilization, HCN, and siderophore production [26].

Nitrogen—an essential component of the living system is required for nucleic acid and protein synthesis. Microbial inhabitants of soil fix atmospheric nitrogen and thereby make it available for plants in the form of ammonia in the nitrogen fixation process. PGPR is capable of fixing nitrogen in both symbiotic (*Azotobacter* spp., *Bacillus* spp., etc) as well as non-symbiotic (free-living diazotrophs, *Azospirillum*) manner [27]. In leguminous plants, nitrogen fixation is assisted by rhizobia which undergoes a significant transformation from free-living to nitrogen-fixing bacteroid residing in the root nodules [28]. In the early 21st century, research was oriented toward the development of commercial inoculants of free-living bacteria, such as *Diazotrophicus* sp., *Azotobacter* sp., and *Azospirillium* sp., as a medium to provide nitrogen in a wider range of plants [29]. Commercial inoculum of *Azospirillium* with *Rhizobium* consortia was found effective in enhancing nodulation and to increase in crop yield [30]. To improve nitrogen uptake, studies are focused on the development of microbes that can facilitate the root systems than finding nitrogen-fixing microbes [31].

The next macronutrient required for the growth and development of plants is phosphorus. Though present in ample amounts in agricultural soils, much of it (30–65%) is present in the non-soluble (organic; phosphates, phosphodiesters, etc) form [32]. As organic forms are not taken up by plants, a significant level of their acquisition is fulfilled by supplementation in the form of fertilizers. Additionally, phosphate-solubilizing bacteria belonging to the genus *Pseudomonas*, *Alcaligenes*, *Bacillus*, *Corynebacterium*, and others are capable of transforming the recalcitrant forms into easily available forms for uptake by plants [32]. Secretion of organic acids or H^+^ ions by phosphate solubilizing bacteria promotes the solubilization of inorganic phosphate that has undergone complex formation with calcium, aluminum, and iron [33]. Similarly, the release of phytase by phosphate-solubilizing bacteria promotes the production of its active form from organic moieties.

HCN—a secondary metabolite produced among Gram-negative bacteria, is a product of the oxidative reaction of glycine catalyzed by HCN synthase [34]. Being a flavoenzyme, its reaction is mediated by flavin adenine dinucleotide (FAD), while pyrrolnitrin acts as an inhibitor of the reaction [35]. Though the production of HCN by microbial population was earlier referred to as a plant protective mechanism, it was later recognized to play an active role in enhancing the availability of phosphorus to plants [36]. Iron—a micronutrient, is considered an essential element in terms of its requirement for the growth and development of plants. Despite its abundance in the earth’s crust, scarcity of the bioavailable iron reported in most soils accounted for its occurrence mostly in the ferrous (Fe^2+^) form [37]. Having greater requirements for ferric (Fe^3+^) form, bacteria secrete iron-binding ligands (referred to as siderophores) to make Fe^3+^ available for bacterial use. Siderophore (Extracellular low molecular weight water-soluble compound of microbial origin) production is preferably associated with active sequestration of iron (Fe^3+^) for its transport into the interior of the cell (ferripyoverdine; Combination of Fe^3+^ with pyoverdin) for use in nitrogen fixation, respiration, and photosynthesis [38]. 

### 3.3. PGPR in Ion Homeostasis and Osmotic Balance

Microbe-mediated siderophore production and pH changes brought about at the rhizospheric plane fulfill the nutritional needs of the plants. In spite of enhancement in the availability and uptake of nutrients, plants are often prone to disturbances that arise due to stress conditions. Under salt stress conditions, an influx of salt at an enhanced rate following nutrient-to-mineral exchange results in a nutritional imbalance in plants. Disturbance in ion homeostasis is often observed in plants that owe the property of being poor excluders of Na^+^ and having a high affinity for Cl^−^ ions [39]. The ions (both Na^+^ and Cl^−^) above their required amount exert toxic effects in terms of reduced growth and senescence following their translocation and accumulation in aerial parts of plants [39]. As only a smaller fraction of Na^+^ undergoes recirculation, the perturbance of the Na^+^ to K^+^ ratio results in the inhibition of the cytosolic activities along with interference in the activities of enzymes associated with respiration and photosynthesis [40]. Microbes reduce the toxic ion uptake through the production of exopolysaccharides (EPS) and regulating the expression of ion affinity transporters. The EPS enables the survival of plants under inhospitable conditions by altering the root structure (developing extensive rhizosheaths) and promoting the trapping of toxic ions in the EPS matrix [18,41,42]. Membrane localized Na^+^/H^+^ antiporter (Salt overlay sensitive channel, SOS1) proceeds with efflux of cytosolic Na^+^, while high-affinity K^+^ transporters (HKT) enhance uptake of K^+^ at the root surface [43]. Additionally, sensing of the salt stress signal by calcineurin B-like protein (CBL4; also known as SOS3) makes it undergo interaction and as such complex formation with CBL-interacting protein kinase (CIPK24, also known as SOS2). Phosphorylation of SOS1 by the SOS2-SOS3 complex results in its activation and, thereby, sustenance of the K^+^-transport for maintaining the Na^+^/K^+^ ratio [44].

Accumulation of the salt ions often causes disturbance in the osmotic balance in plants. PGPR improves the plant-water relationship through enhancement in the production of osmolytes for uptake by plants at the root surface [45]. Stimulation for the uptake of compatible solutes (osmolytes) such as proline, glycine, polyamines, etc., followed by accumulation is essential for maintaining osmotic balance to avoid oxidative damage to the cellular components [46]. The compatible solutes affect hydraulic conductivity towards the regulation of the water potential and stomatal opening. Inoculation of *Bacillus megaterium* causes increased water conductance in maize grown under salt stress via increase in the expression of two plasma membrane aquaporin protein isoforms [47]. Seed inoculation of *Bacillus aquimaris* causes an increase in the total soluble sugar, shoot biomass, and enhanced NPK accumulation, besides causing a reduction in the Na^+^ content of the leaves [48]. PGPR-mediated proline accumulation is often correlated with an increase in the expression of pyrroline-5-carboxylate synthase (*P5CS*), thereby leading to enhancement in the accumulation of free proline in plants [49]. The biofilm formation at the root surface increases stable soil aggregates that often attribute protection of plants against water stress [50].

### 3.4. PGPR Produced Phytohormones and Plant Growth

PGPR-mediated exogenous release of hormones, metabolites, and enzymes modulate plant hormonal balance, thereby influencing their growth under extreme stress conditions. Capable of altering water and nutrient uptake, morphology and metabolism, and tolerance to different environmental stresses, they play a key role in boosting the growth and development of plants [51]. Indole-3-acetic acid (IAA) production is a relatively common trait of PGPR that increases the fitness of plants growing under stress conditions. PGPR utilizes tryptophan present in the root exudate for inducing IAA synthesis at the rhizospheric plane [52]. IAA produced by PGPR exerts a strong effect on root growth and its architecture [52,53]. Co-inoculation of IAA-producing *Azospirillum brasilense* Az39 with *Bradyrhizobium japonicum* E109 causes enhancement in the germination and growth of corn and soybean [53]. Inoculation of Bacillus subtilis GB03 in Arabidopsis regulates the activity of cell wall loosening enzymes, besides promoting growth by having a tight regulation of auxin homeostasis [54]. Similarly, IAA-producing PGPR was found to enhance nutrient uptake under hydroponic conditions and exhibited higher growth of roots and leaves under salinity conditions [55]. PGPR-producing auxin induces a change in the expression of genes pertaining to the cell wall, hormone, and defense-related genes, decreasing stomatal size and density, increasing root biomass, and leading to the activation of auxin response genes associated with the growth and development of plant [56].

Gibberellins are synthesized in the terpenoid pathway and are often found associated with developmental processes such as cell division and elongation, and they have a role in germination following an exit from seed dormancy. PGPR strains such as *Bacillus amyloliquefaciens* RWL1, *Enterococcus faecium* LKE12, and others are known to show the production of gibberellins [57,58]. PGPR-mediated production of gibberellins causes enhancement in the hypocotyl and stem growth; thereby assisting in regulating leaf and root meristem size [59]. Cytokinins represent a group of purine-based plant hormones often found associated with regulation cell division and differentiation of the meristematic tissues, chloroplast maturation, and stomatal conductance [60]. Though cytokinin production is a common trait in PGPR, it influences the endogenous cytokinin pool by inducing its synthesis or altering its homeostasis in plants [61]. As the auxin-to-cytokinin ratio plays important role in determining the fate of a cell, inoculation of cytokinin-producing *B. subtilis* in drought-stressed lettuce plants promotes plant growth via modulation of root-to-shoot signaling [62].

Abscisic acid (ABA)—a stress hormone synthesized in response to stress regulates the expression of genes that impart stress tolerance [18]. Its function is primarily associated with triggering the adaptive response, i.e., the abscission of leaves and retarded shoot growth, with overcoming adverse environmental conditions. Many PGPR such as *B. licheniformis*, *Pseudomonas fluorescens*, *A. brasilense*, and others, are known to show ABA production [63]. PGPR-mediated ABA production modulates ABA biosynthesis and its dependent signaling pathways that cause resistance by mitigating the sensitivity of plants to water scarcity. Its accumulation at the root surface performs the function of enhancing water uptake via an increase in root growth and stimulation of lateral root formation [2]. Under drought conditions, its translocation from root to aerial parts, particularly leaves, causes stomatal closure in order to maintain water balance via a reduction in transpiration activity [64]. PGPR-mediated enhancement in the plant ABA pool relieves plants of the effects of growing under different abiotic stresses.

Ethylene—a gaseous stress hormone effective at extremely low concentrations is produced from its precursor 1-aminocyclopropane-1- carboxylase (ACC) by the ACC oxidase enzyme in plants [2]. PGPR, capable of producing ACC deaminase, reduces ethylene production, thereby enhancing stress tolerance by promoting growth in plants [65]. The ACC deaminase-producing bacteria in the rhizospheric plane degrade ACC from the root exudate, which progresses with a fall in the ACC levels inside the plants. The ACC deaminase converts ACC to ammonia and α-ketoglutarate that keep ethylene at bay from reaching significant levels in order to exert a reduction in plant growth [66].

Jasmonic acid (JA) and Salicyclic acid (SA), having ubiquitous expression in plants, constitute other armories for defense against different stress conditions [67]. They perform definite roles pertaining to different developmental processes such as germination, root and shoot growth, and flowering in plants. PGPR, in particular endophytes, were found capable of synthesizing JA and SA, thereby playing a critical role in regulating the growth and development of plants [68]. JA-mediated induction of the signaling pathways results in the activation of calcium channels that causes enhancement in the levels of cytosolic calcium and by playing an active role in upregulating the expression of genes involved in glutathione (GSH) synthesis for scavenging the ROS as part of the antioxidant response against abiotic stress [68,69]. Inoculation of the plants with *P. fluorescens* Pf4, *B. amyloliquefaciens* LJ02, and *Burkholderia phytofirmans* PsJN results in the enhancement of endogenous levels of SA [58,70,71].

## 4. PGPR in Abiotic Stress Tolerance

Plants, being immobile, are destined to complete their life cycle in one place. It exposes them to extreme conditions that exert definite pressure on plants to evolve and succeed for traits to withstand the fluctuations in the environmental conditions. The changes in the climatic conditions produce aggravated effects that impose serious effects such as disturbance in genetic makeup, perturbed metabolic activities, and impaired crop yield for a majority of plants worldwide [18]. The selection pressure that confronts the growth and development of plants are categorized into biotic (bacteria, virus to grazing by higher animals) and abiotic (heavy metals, extreme temperature, drought up to salinity) factors [18,72]. Abiotic stresses affecting different aspects of plant growth pose a serious threat to the survival of plants [73]. It progresses with a significant reduction in germination and strong effects on photosynthetic activity, carbon assimilation, and on overall crop productivity [74]. Under such circumstantial conditions, the phytomicrobiome came to the forefront to assist in the survival of holobiont (plants in association with microbes) in extreme environmental conditions [16,17]. 

### 4.1. PGPR in Mitigating Heavy Metal Stress

Heavy metals (Mercury, Hg; Arsenic, As; Chromium, Cr; and others) discharged from natural (volcanoes, etc) and anthropogenic (industries, mining, etc) sources result in pollution of the environment [75]. Being recalcitrant to degradation, their persistence in the environment exhibits spatial distribution and, as such higher toxicity across different environmental gradients [76]. Their contamination of the agricultural soils results in their accumulation, and as such, transfer into the food chain and crops, resulting in life-threatening consequences among humans, animals and mankind as a whole [77]. Presence of heavy metals at higher levels in soil results in their uptake and accumulation in different parts of the plant, where they exert serious consequences in terms of interference with the uptake of nutrients, growth parameters via modulation of the enzymatic activities, disruption of the metabolic machinery, etc [77]. Offering a great concern to mankind, their remediation is primarily aimed at physical and chemical clean-up procedures that vary in terms of efficiency and effectiveness [78]. 

Considering their limitations, plant-based remediation strategy commonly referred as phytoremediation offers an advantage of being an environmental friendly, efficient, and cost-effective strategy to achieve the remediation of heavy metals from the soil [77]. Though it offers a greener approach to achieving remediation, plants employed in the process face extensive stress that causes a reduction in proficiency in extracting heavy metals from the contaminated soils [79]. In such a scenario, microbial strains exhibiting wider resistance to heavy metals offer adaptability to plants in their survival in hostile environments and in leveraging their toxic effects on plants by causing precipitation and as such reduction in metals in soils [80]. They exert a positive influence on plant growth and biomass by providing suitable nutrients for uptake, besides playing an important role in converting toxic forms to less toxic ones, production of chelating agents, modification of phytohormone levels, and the production of biofilms [77,78,79,80]. Association of *Bacillus xiamenesis* and *Bacillus gibsonii* with roots of *Sesbania sesban* commonly inhabiting saline soils and waterlogged conditions, enhances growth and phytoextraction potential via the production of ACC-deaminase that cause a decrease in the production of ethylene, besides enhancing the production of exopolysaccharides (EPS), and phytohormones such as indoleacetic acid (IAA) [79]. Inoculation of cadmium (Cd) and lead (Pb) resistant *Bacillus* species (QX8 and QX13) of the *Solanum nigrum* L. roots enhances uptake of nutrients such as phosphorus (P), solubilization of iron (Fe), nitrogen (N_2_) fixation in addition to increasing in the uptake and accumulation of Cd and Pb in aerial parts of the plant [77]. Similarly, inoculation of *Pseudomonas aeruginosa* and *Burkholderia gladioli* to *Lycopersicon esculentum* seedlings results in a lowering of the expression of metal transporter genes with enhancement in the plant growth (shoot and root length) and an increase in the production of photosynthetic pigments such as chlorophyll, carotenoids, and xanthophylls [80].

Information on the plant growth-promoting rhizobacteria employed in mitigating different stresses is summarized in Table 1. The following sub-sections discuss information on the valuable microbial-based resources that are oriented for the upliftment of the constraints towards achieving sustainability in agriculture.

### 4.2. PGPR in Alleviating Effects of Extreme Temperature

Change in climatic conditions has exacerbated the severity of environmental stressors. Having a profound effect on the adaptation and survival of plants, it accounts for a huge loss in overall productivity [18]. Gradual increase in temperature (5–15 °C above the normal) proceeds with a significant effect on the growth and development of plants [135]. The increase in temperature that often proceeds with the decrease in the soil water content led to a significant reduction in the cultivable capacity of the soil i.e., a shift from fertile to marginal [18]. Perceived as an outcome of the climatic conditions, water scarcity adversely affects chlorophyll content and rate of photosynthesis, nutrient acquisition, carbon assimilation, and gaseous exchange at the leaf surface, which is considered critical for the survival of plants [73]. It is well documented that an increase in the temperature in the range of 3–4 °C results in a significant decrease i.e., almost 15–35% in Asia and Africa and by an amount of 25–35% in the middle-east countries. Representing a well-defined heat stress, mechanisms encompassing alteration in the expression of genes encoding heat shock and scavenger proteins, and a trigger for the accumulation of metabolites required for normal cellular metabolism, starts operating as part of the internal defense of plants [103,135].

Though tolerance to heat stress attributed by PGPR largely remains speculative, their ability the produce exogenous polysaccharides attributed it with molecules for use in facilitating their interaction with the root appendages, followed by colonization of roots and in shielding them against desiccation via induction in the formation of biofilms [96]. The exopolysaccharides released into the extracellular matrix provide a range of macromolecules necessary for the growth and development of plants in addition to the interface for improving water uptake through the rhizospheric plane and enhancing its retention by several folds [136]. The ability of *P. putida* to survive in low-moisture soils promotes its colonization of the wheat and sunflower roots via an increase in the soil adhering capacity and in promoting the formation of stable soil aggregates [50]. Inoculation of potato plants with *Burkholderia phytofirmans* promotes enhancement in the root and shoot biomass and an increase in the stem length following their growth at high temperatures [137]. Inoculation of plants with *P. aeruginosa* not only increases its water retention capacity but also contributes to an increase in the total chlorophyll content, length of roots, leaf area, dry matter, and a substantial decrease in the cell membrane injury when grown under high-temperature conditions [138].

Cold stress contributing to slow down or suspension of metabolic activities contributes significantly to a reduction in the yield. Compared to control, inoculation of the explants of *Vitis vinifera* cv. Chardonnay with *Burkholderia phytofirmans* PsJN results in improved tolerance of the plantlets to cold [86]. Holding endophytic colonization of the plantlets reduced their sensitivity to cold, thereby improving their growth at an ambient temperature of 26 °C and at a low temperature of 4 °C. With improved growth and physiological activities, the major benefits were observed in the growth of roots and to overall biomass of the platelets. Bacterization of the wheat results in significant improvement in root and shoot length along with their dry mass, an increase in the free proline, starch, chlorophyll, and anthocyanin contents, and a subsequent decrease in the electrolyte leakage and Na^+^ to K^+^ ratio [128]. In another study, inoculation of wheat with *Pseudomonas* spp. PPERs23 results in an improvement in the nutrient content in shoots that improves grain proportion and thereby overall productivity by 13.4% [139].

### 4.3. PGPR in Alleviating Effects of Drought Stress

A decrease in the soil water level progresses to a change in the land use pattern. In drought, the water potential and turgor pressure are so disturbed that it causes a major hindrance to the flow of nutrients, thereby causing a reduction in the photosynthetic activity [140]. Depending on the duration, drought stress ranges from moderate to prolonged, with implications from interference in the normal cell functions to changes in morphological and physiological traits if prolonged [140]. Suppressing the cellular antioxidant defense, it induces the production of free radicals and reactive oxygen species (ROS) such as superoxide, hydroxyl radical, and hydrogen peroxide, which contributes significantly to the induction of oxidative stress [141]. ROS generation progresses with damage at various levels, such as membrane disintegration and lipid peroxidation, followed by serious damage to proteins and nucleic acids [142]. For growth under drought conditions, plants primarily switch to a drought escape (DE) mechanism that involves growth at a rapid rate and a great reduction in the life span in order to complete the life cycle within the stipulated period of favorable conditions [143]. As part of survival strategies, it proceeds with enhancement in the accumulation of compatible solutes, such as proline, for regulating normal cellular function [144]. In addition to its role in regulating mitochondrial function, proline-mediated activation of stress-responsive genes helps in the regulation of cell proliferation activity. Proline accumulation proceeds with the scavenging of free radicals that decrease the rate of lipid peroxidation, thereby helping to maintain the structural integrity of the membranous structures [145]. In addition to regulation of the physiological function, compatible solutes impart regulation of cellular turgor and stomatal movement towards the regulation of the photosynthetic events that contribute to the overall regulation of growth and development in plants.

Studies performed on the rhizobacteria have suggested their role in alleviating the impact of drought stress that proceeds with enhancement in the growth and overall productivity of the plants [146]. To examine the impact of microbial inoculations, it was found effective in improving nutrient uptake and, as such, the growth of cereals, vegetables, and legumes [40]. PGPR-mediated production of EPS, such as cellulose, and alginate was found effective in mitigating the effects of stress, thereby increasing the tolerance of plants to different environmental stresses [147]. Acting as a barrier around the roots, it regulates bacterial attachment to roots and ensures the movement of water and nutrients toward improving plant growth under drought conditions. PGPR-mediated production of phytohormones adds to the endogenous hormonal pool of plants [148]. Inoculation of plants with IAA-producing bacteria results in increasing root growth via stimulation in lateral root formation and development of root hairs, thereby increasing water and nutrient uptake to cope with the water deficit conditions [120]. IAA-mediated transcription induction of the ACC synthase gene progresses with an increase in the ACC concentration that enhances the production of ethylene. It proceeds with an increase in plant growth mediated by IAA via enhancement in the water uptake and absorption of nutrients [149,150]. Similarly, bacterial isolates belonging to the *Bacillus* genus (DS4 and DS9) capable of growing under drought conditions were found effective in the production of high levels of IAA (1.61µg/mL and 0.9µg/mL) and gibberellins (49.95µg/mL) as part of an attribute in promoting growth under harsh environmental conditions [151]. PGPR-mediated IAA production promotes plant growth and induces stress tolerance via a reduction in the levels of ethylene. Additionally, PGPR plays a beneficial role in drought stress by mitigating the effect of ABA. Foliar spray of *Platycladus orientalis* increases the relative water content and stomatal conductance thereby plays a role in the growth of plants under drought conditions [152].

PGPR-mediated accumulation of osmoprotectants and enhancement in the metabolic activity and antioxidant defense contribute significantly to the promotion of plant growth. Inoculation of maize with *Herbaspirillum seropedicae* and *Azospirillum brasilense* results in improved water usage efficiency, membrane stability, osmolyte accumulation, and enhanced ACC deaminase and antioxidant enzymes activities [92,153]. *Bacillus thuringiensis* inoculation of *Lavandula dentate* overcomes the effect of drought stress via an enhancement in the proline content, nutrient uptake, and increase in metabolic activities [154]. Inoculation of soybean with *Pseudomonas* sp. results in an increase in chlorophyll content, stem height, and fresh weight when grown under water deficit conditions [155]. Inoculation of maize genotype TP30 with *Bacillus* spp. causes a significant increase in membrane integrity via an enhancement in the antioxidant defense with a subsequent decrease in electrolyte leakage [94]. PGPR strains such as *P. jessenii*, *Anthrobacter nitroguajacolicus*, and *P. synxantha* inoculation of rice seedlings, *P. putida* in maize, *B. polymyxa* in tomato, *B. subtilis* in mustard, *Pseudomonas* and *Mesorhizobium cicero* in green gram, are capable of promoting plants grown under drought stress conditions [46,156,157,158]. Application of *E. cloacae* improves the growth of the root system and, as such relative water content for plants grown under water stress conditions [159].

### 4.4. PGPR in the Alleviation of Salt Stress

Soil salinity (electrical conductivity of 4 dSm^−1^) is a parameter attributed to the presence of high levels of salts such as NaCl in addition to MgSO_4_, K_2_SO_4_, MgCl_2_, etc., that originates from weathering, irrigation, and evaporation of shallow groundwater [18]. An increase in salt concentration of soil exposes plants to osmotic (accumulation of salts at roots that hampers uptake of water) and ion (surplus accumulation of Na^+^ in aerial parts of plants especially leaves that cause reduction in photosynthesis and other metabolic activities) stress [160]. Salinity stress causes a decrease in the cultivable land, i.e., almost 1–2% annually, and is projected to show a progressive decrease on account of global climate change [18]. Being deleterious to plant health, it interferes with the physiological processes of plants and exerts a strong impact on important soil parameters such as nitrification, microbial diversity, etc [161]. In addition to its effect on the extraction of water required for optimal growth, it adversely affects flowering and fruiting and causes aberrations to reproductive structures that affect normal cellular functioning and overall yield [18]. Acting as a limiting factor for the growth of salt-sensitive plants and even some halophytes, it causes up to 70% reduction in the yield of some plants such as rice, wheat, maize, and barley [162].

Water scarcity, which encompasses both drought and salinity, presents an unpredictable constraint with a serious impact on morphological, physiological, and biochemical parameters and, thus, on overall plant health [73]. The salt tolerance parameters such as survival, vegetative growth, and harvestable biomass quantified over a period of time are either adopted by inheriting the adaptable genetic traits or through the exclusion of salts at the root surface [124]. In certain plant species, especially halophytes, an enhancement in the conductance of accumulated salts via the xylem to aerial parts, in particular leaves for precipitation or to salt glands (special structures developed in shoots) for excretion at the surface [41]. Additionally, plants develop interactions with the rhizospheric microbiota that range from mutualism to antagonism after successful colonization and establishment at the rhizospheric plane. The prerequisite condition for the successful establishment of PGPR in the rhizospheric region includes the property of being motile and with the ability to adhere to plant roots via pilli or surface localized proteins [18,163]. The root exudates rich in nutrients fulfill the requirement of microbes to flourish while prompting changes such as nutrient availability and defense that elicit strong responses related to stress tolerance and offer a definite mechanism for promoting plant growth [18,120].

An alternative strategy of PGPR makes use of EPS for the successful establishment of bacteria in roots and soil particles, besides playing an intricate role in developing healthy interaction between bacteria of different microbial communities [164]. The application of *Marinobacter lypoliticus* and *B. subtilis* on wheat causes a significant decrease in the effects exerted by salt and drought stress [165]. Inoculation of soybean with *Bradhrhizobium japonicum* 532C and *Chenopodium quinoa* with *Bacillus* sp. MN54 led to significant improvement in plant growth under high (36–400 mM NaCl) salt concentrations [166]. Trehalose—a non-reducing disaccharide resistant to high temperature and acidity-prevents protein aggregation and degradation, thereby imparting definite protection to plants against salinity and drought stress [167]. Inoculation of soybean plants with *P. putida* and tomato plants with *Azospirillum brasilense*, *Chryseobacterium*, *P. chlororaphis*, and *streptomyces* sp. causes a reduction in the ethylene levels and, as such, imparts tolerance to salinity stress [57,168]. In a study, the presence of the *Priestia endophytica* SK1 in soil with fenugreek plant (*Trigonella foenum-graecum* L.) grown was found effective in enhancing nitrogen-fixing ability and improvement in the biosynthesis of trigonelline content [169]. In another study, inoculation of Acinetobacter johnsonii (SUA-14) to maize resulted in enhancement in the enzymatic activity of urease, alkaline phosphatase in soil and a subsequent decrease in catalase (39%), superoxide dismutase (26%) and malondialdehyde (27%); thereby imparts resistance of maize to salinity stress [170]. Though inoculation of *B. megaterium* regulates phosphate solubilization, inoculation of *Pseudomonas* sp. to eggplant, *Bacillus* sp. to alfalfa, *B. licheniformis* to red pepper, *Burkholderia cepacia* to cucumber, *Phyllobacterium* sp. to strawberries and *Enterobacter* sp. to okra were found attributing them property to tolerance salinity stress [57,119,171,172,173]. The PGPR strains were found to play a vital role in improvising the resistance of plants to salinity stress and in fulfilling the requirements towards enhanced growth and development of different plants.

## 5. Conclusions and Future Perspectives

**Figure 3:**
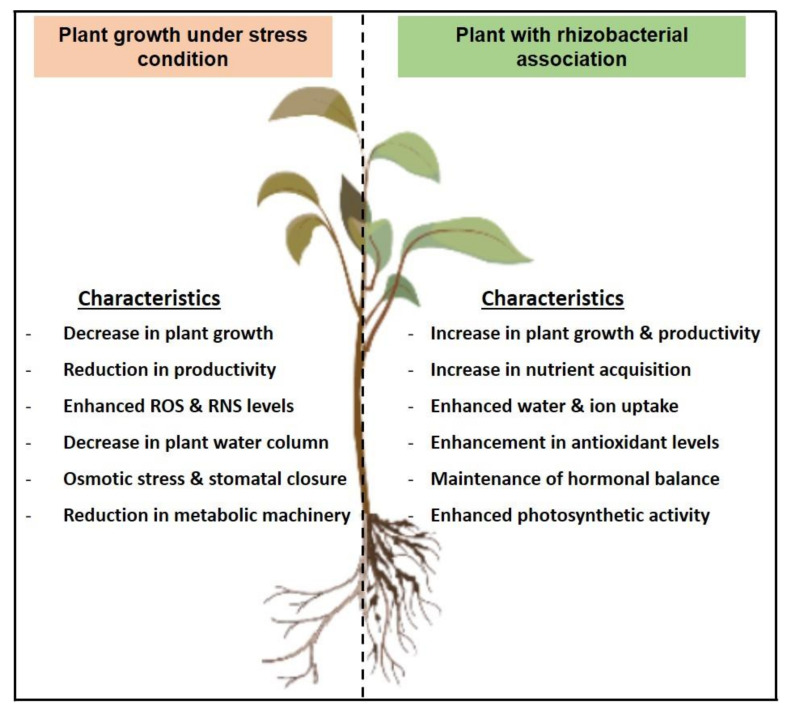
Summarized view of plant-microbiome interactions exerting beneficial effects in plants.

The unabated use of chemical fertilizers in agriculture led to unanticipated environmental impacts that exerted serious health complications. Having an adverse effect on the environment and serious human health concerns, it gained momentum for the development of environmentally friendly alternatives as a suitable replacement for chemical-based fertilizers for use in agriculture (Figure 3). As the development of tolerant cultivars by genetic manipulation encounters various hindrances, the exploitation of soil microbiota for uses as biofertilizers and as biocontrol agents sums up the integral component of the organic farming system. The mechanisms elicited by PGPR include the production of biofilm for enhancing soil binding to roots and regulation of relative water content, triggering of osmotic response via production and accumulation of osmolytes, enhancement in nutrient uptake, and maintenance of ion homeostasis, enhancement in the production of plant growth regulators, improved antioxidant defense, chlorophyll, carbon and nitrogen levels, HCN and siderophore production, besides regulating the expression of stress reliever genes and proteins. It induces a protective system to mitigate the existing stresses in a time-sensitive and cost-effective manner. With the rising emphasis on sustainability, environmental safety, and food security, the employment of bio-inoculants will help to overcome the constraints resulting from a persistent change in the climatic conditions and offer a promising alternative to achieve sustainability in agriculture in an environmentally friendly manner.

The PGPR attributing multiple plant-growth-promoting (PGP) traits to plants increases their resilience to overcome the effects of stresses under prevailing environmental conditions. Since the diversity of PGPRs depends on soil dynamics and plant species, studies should be directed towards delineating the community structure in the rhizospheric plane and elucidating their ecological association and mechanisms thereof that contribute to enhancement in the plant growth and tolerance to different stresses. Additionally, knowledge of the endogenous microbial communities will provide insights into designing the inoculant strains with traits that optimize their performance and maximize their benefits on application under field conditions. Delineating their biochemical and physiological profile under conditions of varying pH, extremes of temperature, drought, and salinity will provide important insights into survival under stress conditions and their dependence on the environment for bio-stimulation and efficacy. It will also provide the baseline data for genetic approaches to engineer bacterial strains that address specificity issues and enhance their ability growth of plants under changing paradigm of existing endogenous microbiota. The futuristic approach should also include mechanistic exploration of the basic architecture of regulatory circuits and harness them for improvement in the yield that could serve the purpose of alleviating stress and achieving sustainability in agriculture to fulfill the global needs for food.

## Figures and Tables

**Table 1:** PGPR mediated promotion of plant growth via, alleviation of abiotic stresses.

Bacterial Strain(s)	Beneficial Effect	Stress Type	Reference
**Abiotic stress (Environmental conditions)**			
*Pseudomonas monteilii*, *Cronobacter dublinensis*, *Bacillus* sp.	Enhanced in nutrient and water uptake	Abiotic stress	[81]
*Achromobacter piechaudi*	Enhancement in fresh and dry weight of plant, reduction in ethylene level	Moisture	[82]
*A. brasilense*	Maintenance of Ion homeostasis	Osmotic stress	[83]
*Azospirillium brasilense* Sp245	Production of phytrochrome	Osmotic stress	[84]
*A. brasilense* Az 39	Enhancement in osmolyte production accumulation	Osmotic stress	[53]
**Temperature stress**			
*Pseudomonas* sp. AMK-P6	Increased expression of stress reliever genes	Heat stress	[85]
*B. phytofirmans* PsJN	Enhancement in ACC deaminase activity	Low temperature	[86]
*P. putida*	Enhancement in ACC deaminase activity	Low temperature	[87]
**Drought**			
*Pseudomonas azotoformans*	Increase in germination rate, shoot and root length	Drought	[5]
*Bacillus* spp.	Enhanced proline content and nutrient uptake, decreased ascorbate peroxidase and glutathione reductase activity	Drought	[88]
*Variovorax paradoxus*, *consortia of Pseudomonas* spp.	Increase in root and shoot length, nutrient uptake, and antioxidant activity, improved biomass	Drought	[89]
*Pseudomonas fluorescens*	Increase in germination rate, improved soil adherence to root tissue dry mass	Drought	[90]
*Rhizobia* spp.	Secretion of volatile compounds and EPS, enhanced cytokinin, and ACC deaminase production	Drought	[91]
*Bacillus subtilis (LDR2)*	Enhanced check of regulatory component (CTR1) of the ethylene signaling pathway	Drought	[92]
*Azosperillum brasilense SP-7*	Increased water uptake and chlorophyl content, improved nitrogen and carbon level, decrease in ethylene and abscisic acid	Drought	[93]
*Azospirillium* spp.	Increase in germination rate and proline content, improved seedling growth and shoot dry weight	Drought	[94]
*Pseudomonas* spp., *Enterobacter* spp., *Bacillus sporothernoduran*	Increase in nutrient uptake, chlorophyl content, plant biomass, and siderophore production	Drought	[95]
*Pseudomonas chlororaphis O6*	Secretion of volatile compounds, osmolyte accumulation, and activity of antioxidants	Drought	[96]
*Klebsiella* sp. *IG3*	Enhancement in ACC deaminase activity	Drought	[97]
*Achromobacter xylosoxidans*, *P. oryzihabitans*, *Variovorax paradoxus*	Increase in auxin production, improved root mass, enhanced ACC-deaminase activity, and decreased ethylene concentration	Drought	[98]
*P. putida* H-2-3	-	Drought	[57]
*Pseudomonas aeruginosa*	Regulation of relative water content, upregulation of stress-responsive genes, Increased root and shoot length	Drought	[57]
*Phyllobacterium brassicacearum*	Enhancement in abscisic acid production	Drought	[99]
*Bacillus pumilu*	Regulation of plant growth; leaf numbers and, size and number of tubers	Drought	[100]
*Bacillus licheniformis* K11	Enhancement in ACC deaminase activity, increased expression of stress-responsive genes	Drought	[101]
*A. brasilense*	Enhancement in osmolyte production and accumulation	Drought	[102]
*Bacillus cereus*, *Bacillus subtilis*, *Serratia* sp.	Enhanced chlorophyl content, root growth, decreased amount of malondialdehyde content	Drought	[103]
*Azospirillum* sp.	Enhancement in lateral root formation, improved uptake of water and nutrients	Drought	[104]
*Bacillus* spp.	Enhancement in osmolyte production and accumulation	Drought	[105]
*Azospirillium* sp.	Improved uptake of water and nutrient	Drought	[106]
*P. putida P45*	Enhancement in EPS production	Drought	[46]
*B. subtilis GB03*	Enhancement in osmolyte production and accumulation	Drought	[107]
*Pseudomonas putida*	Enhanced proline content and water uptake, decrease in production of ROS	Drought	[46]
*Pseudomonas mendocina*	Improvement in antioxidative enzyme production	Drought	[108]
*Paraphaeosphaeria quadriseptata*	Enhancement in the expression of stress reliever genes	Drought	[109]
*B. subtilis*	Volatile compound production	Drought, salinity	[54,110]
*Rhizobium* sp.	Enhancement in EPS production	Drought	[111]
*Paenibacillus polymyxa*	Enhancement in the expression of stress reliever genes	Drought	[112]
**Salinity**			
*Pseudomonas*	Enhancement in IAA production and ACC deaminase activity	Salinity	[113]
*Arthrobacter protophormiae*	Enhancement in ACC deaminase activity	Salinity	[114]
*B. subtilis*	Regulation of ion homeostasis	Salinity	[115]
*Alcaligens* sp., *Bacillus* sp., *Ochrobactrum* sp.	Improved seed germination and seedling growth via enhancement in ACC deaminase activity	Salinity	[116]
EPS-producing bacteria	Enhancement in EPS production	Salinity	[117]
*Pseudomonas pseudoalcaligenes*	Enhancement in osmolyte production and accumulation	Salinity	[118]
*Brevibacterium iodinum*	Enhancement in IAA production and ACC deaminase activity, Increase in ammonia and HCN production	Salinity	[119]
*Pseudomonas aurantiaca*, *P. extremorientalis*	Improved phytrochrome production	Salinity	[120]
*Rhizobium* and *Pseudomonas*	Enhancement in osmolyte production and accumulation	Salinity	[121]
*Sinorhizobium meliloti*	Improvement in antioxidative enzyme production	Salinity	[122]
*Piriformospora indica*	Improvement in antioxidative enzyme production	Salinity	[123]
*Acinetobacter*, *A. faecalis*, *P. aeruginosa*, *B. Cereus*, *Enterobacter hornaechei*, *Pantoae agglomerans*	Enhancement in ACC deaminase activity	Salinity	[124]
*P. fluorescens*	Enhancement in ACC deaminase activity	Salinity	[125]
*Bradyrhizobium japonicum*	Improvement in antioxidative enzyme production	Salinity	[126]
*A. brasilense*	Regulation of ion homeostasis	Salinity	[127]
**Metal stress**			
*Brevundimonas*, *Acaligenes faecalis*	Reduced toxicity and enhancement in phytoremediation potential	Mercury	[128]
*Rhizobium* sp., *Microbacterium* sp.	Reduced toxicity, improvement in nitrogen content	Chromium (VI)	[129]
*Bradyrhizobium japonicum* CB1809	Enhancement in growth and plant biomass	Arsenic	[130]
*Bacillus megaterium*	Reduced toxicity	Lead	[131]
*Bacillus*	Enhancement in phytoremediation potential	Chromium	[132]
*Staphylococcus*, *Aerococcus*	Reduced toxicity	Cadmium, copper, lead and zinc	[132]
*Azotobacter chroococcum* HKN-5, *Bacillus megaterium* HKP-1, *B. Mucilaginosus* HKK-1,	Reduced toxicity and stimulation of plant growth	Lead, Zinc	[133]
*Bacillus subtilis* SJ-101	Enhanced nickel accumulation	Nickel	[134]

## Data Availability

Not applicable.
